# Characterization of combined linagliptin and Y2R agonist treatment in diet-induced obese mice

**DOI:** 10.1038/s41598-021-87539-7

**Published:** 2021-04-13

**Authors:** Henrik H. Hansen, Rikke V. Grønlund, Tamara Baader-Pagler, Peter Haebel, Harald Tammen, Leif Kongskov Larsen, Jacob Jelsing, Niels Vrang, Thomas Klein

**Affiliations:** 1 Gubra, Hørsholm Kongevej 11B, 2970 Hørsholm, Denmark; 2 Department of Cardiometabolic Diseases Research, Boehringer Ingelheim Pharma GmbH & Co., Biberach, Germany; 3 PXBioVisioN, Hannover, Germany

**Keywords:** Pharmacology, Endocrine system and metabolic diseases

## Abstract

Dipeptidyl peptidase IV (DPP-IV) inhibitors improve glycemic control by prolonging the action of glucagon-like peptide-1 (GLP-1). In contrast to GLP-1 analogues, DPP-IV inhibitors are weight-neutral. DPP-IV cleavage of PYY and NPY gives rise to PYY_3-36_ and NPY_3-36_ which exert potent anorectic action by stimulating Y2 receptor (Y2R) function. This invites the possibility that DPP-IV inhibitors could be weight-neutral by preventing conversion of PYY/NPY to Y2R-selective peptide agonists. We therefore investigated whether co-administration of an Y2R-selective agonist could unmask potential weight lowering effects of the DDP-IV inhibitor linagliptin. Male diet-induced obese (DIO) mice received once daily subcutaneous treatment with linagliptin (3 mg/kg), a Y2R-selective PYY_3-36_ analogue (3 or 30 nmol/kg) or combination therapy for 14 days. While linagliptin promoted marginal weight loss without influencing food intake, the PYY_3-36_ analogue induced significant weight loss and transient suppression of food intake. Both compounds significantly improved oral glucose tolerance. Because combination treatment did not further improve weight loss and glucose tolerance in DIO mice, this suggests that potential negative modulatory effects of DPP-IV inhibitors on endogenous Y2R peptide agonist activity is likely insufficient to influence weight homeostasis. Weight-neutrality of DPP-IV inhibitors may therefore not be explained by counter-regulatory effects on PYY/NPY responses.

## Introduction

Defective pancreatic β-cell function is a key pathological hallmark in type 2 diabetes (T2D). Because progressive loss of β-cell insulin secretory capacity is associated with worsening hyperglycemia, targeting intrinsic β-cell activity is considered essential in T2D management. A canonical role of the gut-derived incretin hormone glucagon-like peptide-1 (GLP-1) is potentiation of glucose-stimulated insulin secretion by activation of GLP-1 receptors in islet β-cells. The incretin effect is estimated to account for approximately 50–70% of total insulin secreted following oral glucose administration^1^. Diminished incretin effect is an early characteristic of T2D^2^. In support of the important role of GLP-1 in glucose homeostasis, supraphysiological doses of native GLP-1 can normalize glucose levels and improve insulin sensitivity in T2D patients^3–5^. In addition to glucoregulatory effects, GLP-1 have pleiotropic CNS effects and promotes weight loss by suppressing appetite function ^6^. Active GLP-1 (GLP-1_7–36_) is susceptible to rapid inactivation by the ubiquitous enzyme dipeptidyl peptidase IV (DPP-IV)^7^. Consequently, GLP-1 is effectively degraded upon secretion from enteroendocrine cells and when released into the circulation, resulting in GLP-1_7–36_ having a circulatory half-life of approximately 1 min^7,8^.

While the extremely short half-life prevents the therapeutic use of GLP-1_7–36_, incretin-based therapies have established a foothold in T2D management through the introduction of oral DPP-IV inhibitors and injectable long-acting GLP-1 receptor agonists ^9^. Several DPP-IV inhibitors (‘gliptins’) have been approved for T2D management^10^. The classical therapeutic mechanism for DPP-IV inhibitors is prevention of GLP-1 degradation which promotes two to threefold increases in post-prandial plasma GLP-1_7–36_ levels, leading to improvement of glucose homeostasis due to combined insulinotropic effect and suppression of glucagon secretion^11^. Local inhibitory effects on intestinal GLP-1 degradation may potentially contribute to the glycemic effects of DPP-IV inhibitors^12^. Another incretin, glucose-dependent insulinotropic polypeptide (GIP), is also a substrate for DPP-IV. While the insulinotropic effect of GLP-1 in T2D patients is relatively more preserved compared to GIP^4,13,14^, several lines of evidence suggests that prevention of GIP degradation may potentially contribute to the antihyperglycemic effects of DPP-IV inhibitors. For example, combined GLP-1 and GIP receptor deficiency is necessary to fully eliminate the glucose-lowering action of DPP-IV inhibition in mice^15^, β-cell responsiveness to both GLP-1_7–36_ and GIP is increased in T2D patients with improved glycemic control^16^, and DPP-IV inhibition can partially restore the insulinotropic effect of GIP in T2D patients^17^.

Unlike long-acting GLP-1 receptor agonists, DPP-IV inhibitors do not promote weight loss^18^. Weight neutrality of DPP-IV inhibitors has been explained by increases in circulating GLP-1 concentrations being insufficient to facilitate centrally mediated satiety responses. However, other regulatory peptides involved in energy and weight homeostasis qualify to be DPP-IV substrates^19^. NPY-family peptides such as peptide YY (PYY, i.e. PYY_1–36_) and neuropeptide Y (NPY, i.e. NPY_1–36_) are of particular interest because DPP-IV catalytic rates for these peptides are higher than those for GLP-1_7–36_ and GIP^20^. While NPY is expressed at all levels of the gut-brain axis, PYY is almost exclusively expressed in enteroendocrine cells and co-secreted with GLP-1 in response to nutrient ingestion^21,22^. In contrast to loss of insulinotropic GLP-1 and GIP-1 activity, DPP-IV induced degradation of PYY and NPY changes the receptor affinity and biological action of these peptides. Full-length NPY and PYY bind to Y receptors with little subtype selectivity. While Y1 receptors (Y1R) play a dominant role in PYY_1–36_ and NPY_1–36_ induced feeding upon central administration^23–25^, cleavage by DPP-IV gives rise to NPY_3–36_ and PYY_3–36_ which are high-affinity agonists for the Y2 receptor (Y2R) that mediate appetite suppression and weight loss^26,27^. It has therefore been proposed that DPP-IV inhibitors are weight-neutral as the appetite-suppressive effects of enhanced GLP-1 activity may potentially be counterbalanced by accumulation of intact PYY/NPY resulting in attenuation of anorectic Y2R signaling^11^.

To test this hypothesis, the present study aimed to determine whether combined pharmacological DPP-IV inhibition and Y2R stimulation would unmask potential weight loss promoting effects of the DPP-IV inhibitor. To this end, we administered linagliptin (Tradjenta) together with a Y2R-selective PYY_3–36_ analogue in diet-induced obese (DIO) mice and characterized effects on food intake, body weight and glucose homeostasis.

## Materials and methods

### Animals

The Danish Animal Experiments Inspectorate approved all experiments which were conducted using internationally accepted principles for the use of laboratory animals (license #2013-15-2934-00784). The study was carried out in compliance with the ARRIVE guidelines. 99 C57BL/6J mice were from Janvier Labs (Le Genest Saint Isle, France) and housed in a controlled environment (12 h light/dark cycle, lights on at 3 AM, 21 ± 2 °C, humidity 50 ± 10%). Each animal was identified by an implantable subcutaneous microchip (PetID Microchip, E-vet, Haderslev, Denmark). Mice arrived at 5 weeks of age and were made diet-induced obese (DIO) by offering ad libitum access to tap water and high-fat diet (5.15 kcal/g; 60 kcal-% fat, 20 kcal-% carbohydrate, 20% kcal-% protein; D12492, Ssniff, Soest, Germany) for 26–27 weeks before study start. The mice were kept on the diet throughout the study.

### Drug treatment

DIO mice were randomized into individual treatment groups (n = 9–10 per group) based on body weight measured 3 days prior to initiation of drug treatment. Vehicle was phosphate-buffered saline added 5% mannitol. In study 1, linagliptin monotreatment (3 mg/kg/day, s.c.) was characterized over a dosing period of 14 days. The applied linagliptin dose and treatment time is within the ranges reported to obtain robust DPP-IV inhibition and significant glycemic effects in DIO rodent models^28–30^. In study 2, linagliptin (3 mg/kg/day, s.c.) was administered alone or in combination with an Y2R-selective PYY_3–36_ analogue (compound 38; 3 or 30 nmol/kg/day, s.c.)^31^ for 14 days followed by 6 days of wash-out (no treatment). All drugs were administered once daily at 1 h before lights off. The first dose was given on day 0. Body weight and food intake was monitored daily during the treatment period.

### Oral glucose tolerance test

In study 2, an oral glucose tolerance test (OGTT) was performed one week prior to treatment start (day -7) and on treatment day 12. Animals were fasted for 4 h prior to OGTT. At t = 0 an oral glucose load (2 g/kg glucose; 200 mg/mL, Fresenius Kabi, Sweden) was administered via a gastrically placed tube. Blood samples for measuring blood glucose were collected from the tail vein at t = 0, 15, 30, 60, 120, 180, 240 min. A pre-OGTT blood sample was collected at − 60 min (OGTT on day − 7) and − 240 min (OGTT on treatment day 12). Glucose area under the curve (AUC) calculations were determined as total AUC from the sampling period of 0 to 240 min.

### Blood biochemistry

Terminal cardiac blood samples were collected in EDTA-coated tubes with (for GLP-1 and GIP analysis) or without (for DPP-IV enzyme analysis) 10 µl DPP-IV inhibitor/ml blood (Millipore, Copenhagen, Denmark) prior to sampling. Plasma was separated by centrifugation (3000 × *g* for 10 min at 4 °C) and immediately frozen on dry ice and stored at − 80 °C until further analysis. DPP-IV enzyme catalytic activity was determined as reported previously ^32^. Active GLP-1 and GIP levels were assessed by ELISA (#K150JWC, MesoScale Discovery, Rockville, MD; #27702, IBL, Fujioka, Japan) according to the manufacturer’s instructions. Plasma levels of insulin were measured by ELISA (#K152BZC, MesoScale Discovery, Rockville, MD), according to the manufacturer’s instructions.

### Linagliptin exposure

Linagliptin concentrations in heparinized plasma samples were determined by LC–MS/MS analysis as described previously^33^. Linagliptin exposure was determined in plasma samples before (pre-dosing, i.e. approximately 24 after the previous dose, study 1) and after administration of the last dose (4 h post-dosing) on treatment day 14 (study 1 and 2).

### In vitro assessment of linagliptin effect on NPY and PYY degradation

Human plasma was used as source of endogenous DPP-IV. Healthy adult individuals were enrolled after they provided written informed consent. The local Ethics Committee at the Hannover Medical School approved the protocol. All methods were carried out by observing the applicable legal provisions, including data protection regulations, as well as the principles of medical and professional ethics as laid down in the Declaration of Helsinki issued by the World Medical Association and the ICH/EU recommendations Note for Guidance on Good Clinical Practice (GCP recommendations). Blood samples were collected from the cubital vein into blood collection tubes (EDTA-plasma). Immediately after withdrawal, plasma was separated from cells by a two-step centrifugation procedure (10 min at 2000 × *g* followed by 15 min at 2500 × *g*, both centrifugation steps at room temperature). Plasma aliquots were transferred into 2 ml vials and stored − 80 °C. 30 pmol of human PYY_1–36_ or NPY_1–36_ was incubated in 85 µl PBS, and reaction was started by adding 5 µl plasma with or without 1 µM linagliptin and incubated for 8 h (NPY) or 18 h (PYY) at 37 °C. Parent NPY, PYY and corresponding fragments were determined by matrix-assisted laser desorption/ionization time-of-flight mass spectrometry (MALDI-TOF–MS) as described previously^34^.

### In vitro assessment of PYY_3–36_ analogue effect on NPY and PYY degradation

NPY and PYY were incubated in human EDTA-plasma in presence or absence of linagliptin and/or Y2R-selective PYY_3–36_ analogue and subsequently subjected to MALDI-TOF–MS analysis to determine the rate of *N*-terminal cleavage by DPP-4. PYY (30 pmol) and NPY (30 pmol) were solubilised in PBS and added to 5 µl human plasma with or without linagliptin (1 µM) and/or Y2R-selective PYY_3–36_ analogue at different concentrations (30–30,000 fmol). The plasma was then incubated for 18 h at 37 °C. Subsequently the reaction was stopped, prepared via solid-phase extraction and subjected to mass spectrometry. After measurement, signal intensities were exported and conversion rates for NPY and PYY were expressed as product/(product + substrate). Statistical significance against controls was tested using Welch’s t-test. Each incubation was carried out in duplicate and measured in duplicate at two different concentrations (n = 8). Data were pooled from three individual experiments.

### Statistics

Data were subjected to relevant statistical analyses using GraphPad v8.4 software (GraphPad, La Jolla, CA). Results are presented as mean ± S.E.M. (standard error of the mean). Statistical evaluation of the data was carried out using an unpaired t-test (plasma analytes in study 1, in vitro data), paired t-test (plasma linagliptin), two-way analysis of variance (ANOVA) with Dunnett’s post-hoc test (body weight, food intake, OGTT), or Kruskal–Wallis test (plasma analytes in study 2). A p-value less than 0.05 was considered statistically significant.

## Results

### Linagliptin promotes marginal weight loss while significantly increasing circulating active GLP-1 and GIP levels

**Figure 1:**
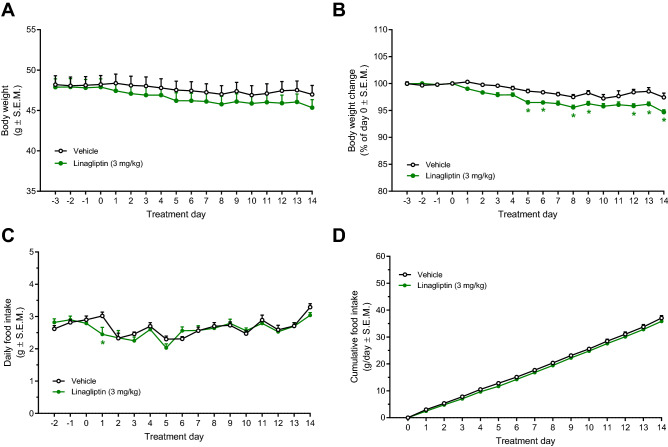
Linagliptin promotes marginal weight loss without influencing high-fat diet intake in DIO mice. *p < 0.05 vs. vehicle controls (two-way ANOVA with Dunnett’s post-hoc test).

**Figure 2:**
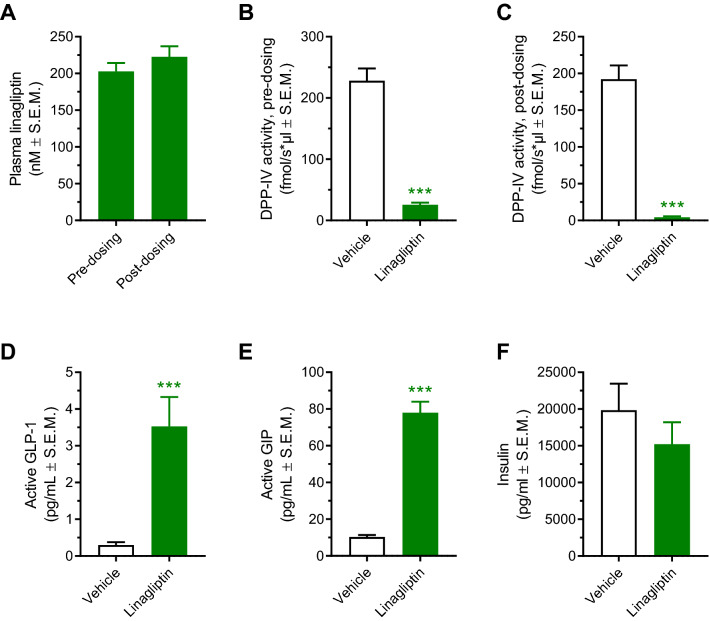
Once daily linagliptin administration for 14 days results in sustained systemic linagliptin exposure, almost complete inhibition of plasma DPP-IV activity and significantly elevated plasma concentrations of active GLP-1 and GIP in DIO mice. **(A)** Plasma linagliptin concentrations before (pre-dosing, i.e. approximately 24 h after the previous dose) and 4 h after the last dosing (post-dosing) on treatment day 14, paired t-test (p > 0.05). **(B)** Plasma DPP-IV activity before the last dose of linagliptin (pre-dosing, i.e. approximately 24 h after the previous dose). **(C)** Plasma DPP-IV activity determined 4 h after the last dosing (post-dosing). **(D)** Plasma levels of active GLP-1. **(E)** Plasma levels of active GIP. **(F)** Plasma levels of insulin. Plasma GLP-1, GIP and insulin levels were measured determined 4 h after the last dosing. ***p < 0.001 vs. vehicle control (unpaired t-test).

In order to obtain maximal DPP-IV inhibition, DIO mice were administered linagliptin using a dose of 3 mg/kg/day (s.c.). Linagliptin monotreatment was initially characterized for effects on key metabolic parameters in DIO mice. Compared to vehicle controls, linagliptin promoted a marginal weight loss (2.8%, p < 0.05) following 14 days of treatment (Fig. 1A,B). Food intake was essentially unaffected by linagliptin treatment (Fig. 1C,D). Linagliptin treatment once daily resulted in high linagliptin exposure (~ 200 nM), as measured in plasma samples before (approximately 24hrs after the previous dose) and 4 h after administration of the last dose on treatment day 14 (Fig. 2A). At both sampling times, almost complete inhibition of plasma DPP-IV catalytic activity was observed (Fig. 2B,C). Correspondingly, linagliptin treatment significantly elevated plasma levels of active GLP-1 (12-fold increase) and GIP (8-fold increase), as measured 4 h post-dosing (Fig. 2D,E). Terminal plasma insulin levels were unaffected by linagliptin treatment (Fig. 2F). Except for significantly reduced resistin and TNF-α concentrations, other plasma markers, including total PYY and pancreatic polypeptide, were unaffected by linagliptin treatment (Supplementary Fig. 1).

### Linagliptin inhibits conversion of NPY and PYY in vitro

**Figure 3:**
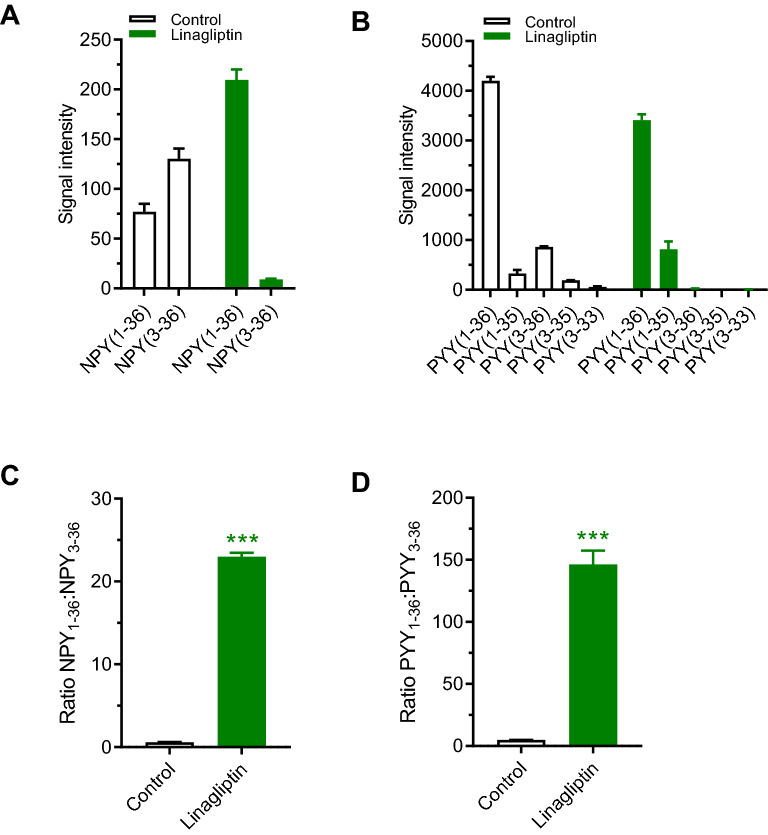
Linagliptin prevents conversion of full-length NPY and PYY to NPY_3–36_ and PYY_3–36_ in human plasma samples. Synthetic NPY_1–36_ and PYY_1–36_ was evaluated as DPP-IV substrates with or without addition of linagliptin [1 µM, incubation times: 8 h (NPY), 18 h (PYY)]. **(A)** Concentrations of full-length NPY_1–36_ and NPY_3–36_. **(B)** Concentrations of full-length PYY_1–36_ and PYY_3–36_. **(C)** NPY_1–36_:NPY_3–36_ ratio, **(D)** PYY_1–36_:PYY_3–36_ ratio. ***p < 0.001 vs. vehicle control.

Synthetic NPY_1–36_ and PYY_1–36_ was evaluated as DPP-IV substrates in human plasma with or without addition of linagliptin. Degradation of NPY and PYY was relatively slow. After 8 h approximately two-thirds of NPY was processed by DPP-IV. In contrast, 30%-50% of PYY was processed after 18 h of incubation. In control samples, NPY_3–36_ was the principal NPY fragment (Fig. 3A). Only small quantities of *C*-terminal processed NPY entities were detectable (data not shown). In contrast, PYY was subjected to both *N*- and *C*-terminal cleavage yielding several peptide fragments, with PYY_3–36_ being the predominant fragment (Fig. 3B). *N*-terminal processing of PYY and NPY was significantly prevented by linagliptin as demonstrated by accumulation of NPY_1–36_, PYY_1–36_ and PYY_1-35_ (Fig. 3A,B). Compared to control, linagliptin shifted ratios of NPY_1–36_:NPY_3–36_ from 0.6 to 23 (Fig. 3C) and PYY_1–36_:PYY_3–36_ from 5 to 146 (Fig. 3D).

### Linagliptin does not augment Y2R agonist-induced food intake inhibition and weight loss

**Figure 4:**
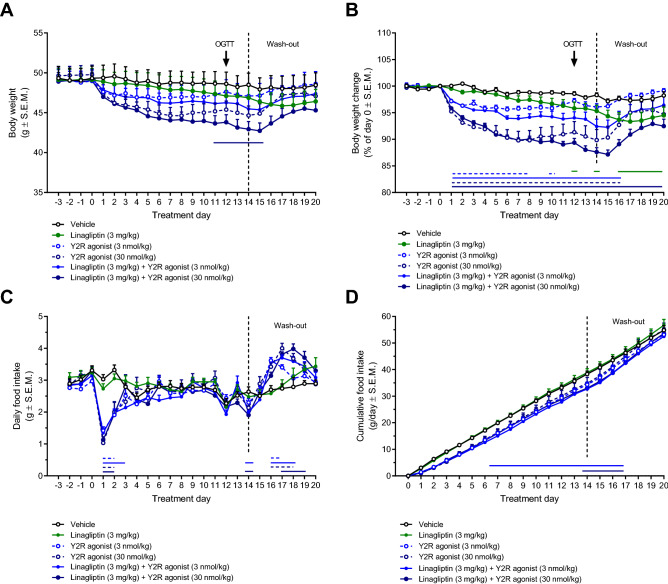
Linagliptin does not augment Y2R receptor agonist-induced food intake inhibition and weight loss in DIO mice. Linagliptin (3 mg/kg, SC) and long-acting Y2 receptor agonist (PYY analogue, 3 or 30 nmol/kg, SC) was administered once daily for 14 days followed by 6 days of wash-out (no treatment) before study termination. **(A)** Absolute body weight. **(B)** Body weight change (relative to day 0). **(C)** Daily food intake. **(D)** Cumulative food intake. Horizontal lines denote significant change in body weight compared to vehicle control (p < 0.05, two-way ANOVA with Dunnett’s post-hoc test). Compared to Y2R receptor agonist monotreatment, combined linagliptin and Y2R receptor agonist administration did not promote further changes in body weight and daily food intake (p > 0.05, two-way ANOVA with Dunnett’s post-hoc test)**.** Compensatory overeating was observed following treatment cessation.

To investigate potential implications of linagliptin-induced bidirectional regulation of GLP-1 and neuropeptide Y-family peptides, we characterized the effect of a Y2R-selective agonist in DIO mice with or without co-administration of linagliptin. Weight curves are depicted in Fig. 4A,B. Compared to baseline, linagliptin monotreatment (3 mg/kg, s.c.) promoted a marginal vehicle-subtracted weight loss (vehicle-subtracted weight loss, − 3.0 ± 1.1%, p = 0.019 vs. vehicle) on the last day of dosing (day 14). Whereas the lowest dose of the PYY analogue (3.0 nmol/kg) showed no statistically significant effect on body weight (vehicle-subtracted weight loss on day 14, − 2.2 ± 0.6%, p = 0.06 vs. vehicle), the highest dose (30 nmol/kg) promoted a robust weight drop (vehicle-subtracted weight loss on day 14, − 8.5 ± 2.0%, p = 0.001, vs. vehicle). Linagliptin + Y2R agonist (3 nmol/kg) co-treatment promoted a slightly greater weight loss than corresponding Y2R agonist monotreatment (vehicle-subtracted weight loss on day 14, − 5.9 ± 1.3%; p = 0.001 vs. vehicle; p = 0.007 vs. PYY analogue 3 nmol/kg), but not when compared to linagliptin monotreatment (p = 0.07). Linagliptin + Y2R agonist (30 nmol/kg) promoted no further weight loss compared to Y2R agonist monotreatment (vehicle-subtracted weight loss on day 14, − 10.7 ± 0.8%, p = 0.001 vs. vehicle; p < 0.001 vs. linagliptin; p = 0.41 vs. PYY analogue 30 nmol/kg). The body weight lowering effect of Y2R agonist mono- and combination treatments gradually wore off after the last day of administration. Weight loss following linagliptin dosing appeared maximal during the wash-out phase (vehicle-subtracted weight loss on day 16–20, 3.5–4.0%; p < 0.05 vs. vehicle). Linagliptin did not influence food intake in DIO mice. In comparison, Y2R agonist treatment suppressed food intake during the first days of dosing (Fig. 4C,D). Compensatory overeating was observed following treatment cessation (Fig. 4C,D).

### Equivalent improvement in oral glucose tolerance following linagliptin monotreatment and combined linagliptin-Y2R agonist administration

**Figure 5:**
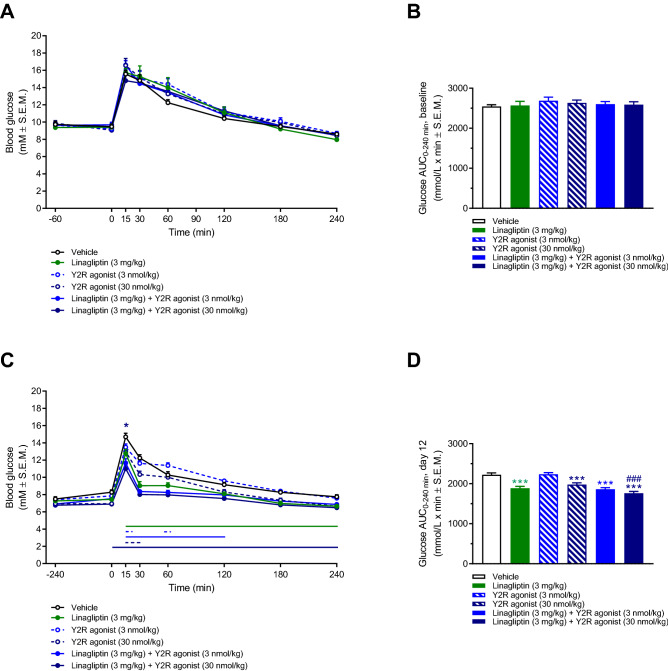
No synergistic effects of linagliptin and Y2 receptor agonist treatment on oral glucose tolerance in DIO mice. An oral glucose tolerance test (OGTT) was performed one week before treatment start (day − 7, **A,B**) and on treatment day 12 (**C,D**). Horizontal lines denote significant change in blood glucose concentrations compared to vehicle control (p < 0.05, two-way ANOVA with Dunnett’s post-hoc test). *p < 0.05, linagliptin (3 mg/kg) + Y2R agonist (30 nmol/kg) vs. linagliptin (3 mg/kg), two-way ANOVA with Dunnett’s post-hoc test.

Oral glucose tolerance was determined one week prior to treatment start (day − 7) and on treatment day 12. On day − 7, fasted blood glucose levels (t = 0) were similar in all DIO mouse groups (group mean 9.1–9.7 mM, p > 0.05) as well as glucose excursion curves and AUC-glucose values (Fig. 5A,B). Compared to day -7, fasted blood glucose levels were significantly lower in all treatment groups (p < 0.05, paired t-test), including vehicle controls, on treatment day 12 (group mean 6.9–8.2 mM, Fig. 5C). Compared to vehicle dosing, linagliptin significantly improved glucose tolerance. Y2R agonist treatment also improved glucose tolerance, albeit with less robust effect on glucose excursions. Improvements in glucose tolerance following combined linagliptin and Y2R agonist administration were superior to Y2R agonist, but not linagliptin, monotreatment (Fig. 5C,D).

### Linagliptin-induced DPP-IV inhibition is unaffected by co-administration of a Y2R-selective agonist

**Figure 6:**
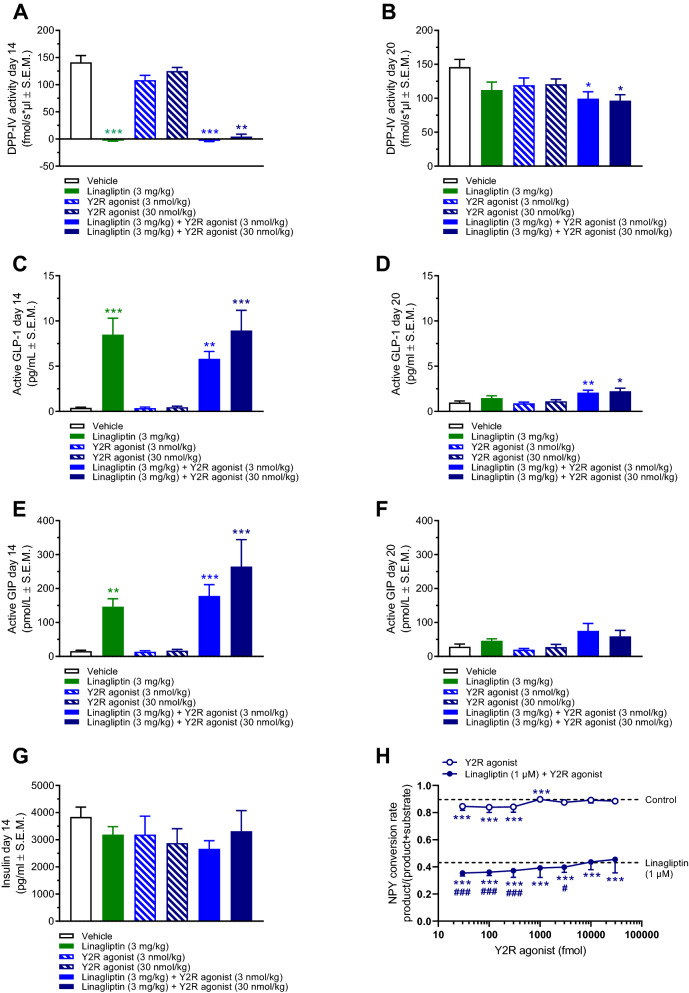
Linagliptin-induced DPP-IV inhibition is unaffected by Y2R agonist co-administration. Linagliptin (3 mg/kg, SC) and long-acting Y2 receptor agonist (PYY analogue, 3 or 3 nmol/kg, SC) was administered once daily for 14 days followed by 6 days of wash-out (no treatment) before study termination. **(A)** Plasma DPP-IV activity measured on treatment day 14 (4 h post-dosing). **(B)** Terminal plasma DPP-IV activity measured on study day 20. **(C)** Plasma active GLP-1 levels measured on treatment day 14 (4 h post-dosing). **(D)** Terminal plasma active GLP-1 levels measured on study day 20. **(E)** Plasma active GIP levels measured on treatment day 14 (4 h post-dosing). **(F)** Terminal plasma active GIP levels measured on study day 20. *p < 0.05, **p < 0.01, ***p < 0.001 vs. vehicle control (Kruskal–Wallis test). **(G)** Plasma insulin levels measured on treatment day 14 (4 h post-dosing). **(H)** NPY conversion rate in human EDTA-plasma added linagliptin (1 µM), Y2 receptor agonist (30–30,000 fmol) or linagliptin (1 µM) + Y2R agonist (30–30,000 fmol). ***p < 0.001 vs. control, ^#^p < 0.05, ^###^p < 0.001 vs. linagliptin alone (Welch’s t-test).

The selected dose of linagliptin (3 mg/kg) completely suppressed plasma DPP-IV activity as measured 4 h post-dosing on the last dosing day (day 14, Fig. 6A). All linagliptin-treated groups showed equally elevated plasma levels of active GLP-1 and GIP on treatment day 14 (Fig. 6C,E). Residual DPP-IV inhibitory activity was detected 6 days after the last linagliptin dose (day 20), being most consistent for linagliptin-Y2R agonist co-treatment groups (Fig. 6B). In the treatment combination groups, this was also reflected by slight, however significantly, increased active GLP-1, but not GIP, levels on day 20 (Fig. 6D,F). The Y2R agonist had no effect on DPP-IV activity as well as active GLP-1 and GIP levels. Treatments did not influence plasma insulin levels (Fig. 6G). Because linagliptin and Y2R agonist co-administration demonstrated stronger inhibitory effects on DPP-IV activity as compared to linagliptin administration alone, we investigated whether the Y2R agonist influenced DPP-IV activity in human EDTA-plasma. In the low fmol range (30–300 fmol), the Y2R agonist slightly reduced NPY conversion rate. Also, linagliptin and the Y2R agonist showed additive inhibitory effects on NPY conversion rate (Fig. 6H). PYY conversion rate was also slightly reduced by the Y2R agonist alone (30–1000 fmol), but unaffected by combined Y2R agonist and linagliptin application (data not shown). At higher Y2R agonist amounts (≥ 1000 fmol), the inhibitory effect of NPY/PYY conversion inhibition was diminished or absent, which was ascribed to reduced solubility of the Y2R agonist.

## Discussion

The present study characterized the metabolic effects of linagliptin with or without co-administration of a Y2R-selective PYY_3–36_ analogue in DIO mice. The linagliptin dose (3 mg/kg, s.c.) promoted complete plasma DPP-IV inhibition (> 98%) maintained throughout the dosing interval. The subcutaneous dosing regimen results in more pronounced DPP-IV inhibition as compared to oral dosing in DIO mice (~ 80% inhibition)^28^. In the clinic, ≥ 80% DPP-IV inhibition by linagliptin administration is sufficient to improve glucose tolerance^35^. Sustained increases in circulating incretin levels were observed in DIO mice treated with linagliptin. Although both linagliptin and the Y2R-selective PYY_3–36_ analogue improved glucose tolerance and Y2R receptor agonist treatment promoted significant weight loss, no synergistic metabolic effects were observed following combined treatment. Therefore, our study in DIO mice indirectly suggests that weight-neutrality of DPP-IV inhibitors is not explained by reduced endogenous Y2R agonist action, and lends further support to the notion that increasing circulating GLP-1 levels by DPP-IV inhibition is insufficient to promote satiety responses.

DPP-IV inhibition is a therapeutic option to extend circulating half-life of GLP-1 and GIP and improve glycemic control in patients with T2D. DPP-IV inhibitors are generally weight-neutral^18^. Our preclinical study corroborates end extends previous studies on linagliptin administration in DIO mice^28,30,36^, by demonstrating that linagliptin induces marginal weight loss when applying a dosing regimen that promotes sustained linagliptin exposure, nearly complete inhibition of plasma DPP-IV activity and ≥ 10-fold increases in plasma GLP-1 and GIP levels. The mild weight loss attained by linagliptin administration in DIO mice may potentially associate to beneficial effects on white adipose tissue mass, liver fat and thermogenesis^28,30^.

Weight-neutrality of DPP-IV inhibitors contrasts the weight loss efficacy of long-acting GLP-1 analogues which are DPP-IV resistant and have slow elimination kinetics^37,38^. It has therefore been speculated that DPP-IV inhibitors are weight-neutral by regulating the biological activity of other peptide hormones involved in energy homeostasis^11^. NPY-family peptides may be potential candidates for promoting metabolic counter-regulatory responses to DPP-IV inhibitor treatment. DPP-IV shows greater affinity for PYY and NPY at physiological concentrations as compared to GLP-1 and GIP^20^. Studies on DPP-IV substrates other than GLP-1 and GIP are limited. Because reliable techniques are lacking to distinguish between intact and cleaved PYY and NPY immunoreactive forms there is little information on the distribution of circulating PYY and NPY cleavage products following DPP-IV inhibitor treatment. We therefore used MALDI-TOF–MS to determine PYY/NPY isoforms in human plasma following incubation with synthetic human full-length NPY and PYY. Linagliptin prevented *N*-terminal cleavage of PYY and NPY resulting in substantial lowering of PYY_3–36_ and NPY_3–36_ concentrations. Consistent with previous studies^39,40^, PYY_3–36_ and NPY_3–36_ appeared to be predominant PYY/NPY fragments generated by DPP-IV catalytic activity in human plasma. Correspondingly, PYY_3–36_ and NPY_3–36_ represent the predominant circulating NPY/PYY isoforms^19,41,42^. PYY_3–36_ is a potent anorectic peptide which activates anorexigenic neurocircuits by stimulating vagal and hypothalamic arcuate Y2R signaling^43–45^. The functional relevance of DPP-IV mediated PYY cleavage is further emphasized by the absence of anorectic effect of exogenous PYY_1–36_ in rats carrying an inactivating DPP-IV point mutation^46^. Sitagliptin has been demonstrated to increase plasma PYY_1–36_:PYY_3–36_ ratios in T2D patients, indicating that PYY is a physiological substrate for DPP-IV^47^. NPY is a neuronal-derived peptide that regulates many aspects of energy balance, including feeding behavior end energy expenditure^48^. NPY_1–36_ is an orexigenic neuropeptide which acts by stimulating hypothalamic Y1R signaling^25^. The anorexigenic actions of PYY_3–36_ are thought partially mediated by inhibiting arcuate NPY neuronal activity^49^. The *N*-terminal tyrosine residue of NPY/PYY is critical for efficient Y1R binding ^50^. As for PYY_3–36_, NPY_3–36_ has markedly reduced Y1R affinity but retained anorexigenic Y2R agonist activity^51^. In contrast to NPY, *C-*terminally truncated PYY variants were detected in human plasma which supports the notion that PYY is a substrate for several endopeptidases ^52,53^. *C*-terminal cleavage renders the peptide Y2R inactive^54^. While our in vitro study demonstrates that linagliptin-induced inhibition of DPP-IV activity prevents conversion of synthetic full-length NPY/PYY to NPY_3–36/_PYY_3–36_, further studies are required to confirm if DPP-IV inhibitors can also modulate endogenous circulating levels of NPY_3–36/_PYY_3–36_.

It is currently unknown if modulation of PYY and NPY biological activity may contribute to shape the therapeutic effects of DPP-IV inhibitors. Because PYY_3–36_ and NPY_3–36_ signaling converge at the level of Y2R function, it may be hypothesized that DPP-IV inhibitors are weight-neutral because lowering of circulating PYY_3–36_ and NPY_3–36_ levels shifts PYY/NPY activity towards Y1R signaling. As a consequence, food intake inhibitory responses subsequent to DPP-IV induced increases in active GLP-1 levels could be counterbalanced by lowered Y2R anorectic activity relative to Y1R orexigenic activity. We therefore sought to determine if pharmacological stimulation of Y2R activity could unmask potential contributory weight-lowering effects of linagliptin in DIO mice. The Y2R-selective agonist (compound 38) was selected based on a patent, demonstrating that compound 38 was one of the most efficacious PYY_3–36_ analogues on food intake inhibition in *db/db* mice^31^. In the current study, food intake suppression was only observed during the first days of Y2R agonist administration while body weight continued to drop over the 14-day treatment period. Sustained weight loss by Y2R agonist treatment may be potentially explained by contributory effects from increased lipolysis and energy expenditure^55,56^. Linagliptin co-administration did not enhance the anorectic response or weight loss attained by Y2R agonist treatment. Following cessation of drug treatment, DIO mice showed compensatory overeating and gradually resumed baseline body weight. The rate of weight regain was different in linagliptin and Y2R agonist treated mice which is likely explained by different pharmacokinetics. Co-administration of linagliptin and the Y2R agonist resulted in slightly stronger inhibitory effects on DPP-IV activity as compared to linagliptin administration alone. This effect likely explains the slightly higher plasma active GLP-1 levels observed in DIO mice receiving combination treatment. Additive DPP-IV inhibitory effects of linagliptin and the PYY_3–36_ analogue were confirmed in vitro. Because NPY was added in 100-fold excess in comparison to the PYY_3–36_ analogue, substrate inhibition seems less plausible. Therefore, future studies must aim to address the underlying mechanism for DPP-IV inhibition conferred by the PYY_3–36_ analogue.

Our findings contrast mounting evidence from pharmacological studies suggesting additive/synergistic effects of GLP-1_7–36_ and PYY_3–36_. In infusion studies, the combination of PYY_3–36_ and GLP-1 confers stronger suppression of food intake and hunger sensation compared to monotreatment in healthy and obese subjects^57–59^. Correspondingly, preclinical studies have demonstrated synergistic anorectic effects and robust weight loss following administration of a GLP-1 analogue in combination with native PYY_3–36_ or PYY_3–36_ analogue treatment^60–63^. Compared to the marked metabolic effects of pharmacological administration of GLP-1 and PYY_3–36_, weight-neutral metabolic effects of DPP-IV inhibitors may therefore result from the modest changes in circulating levels of peptide hormones involved in appetite regulation and weight homeostasis. Both linagliptin and the Y2R agonist significantly improved oral glucose tolerance in DIO mice. As for body weight, combined linagliptin and Y2R agonist treatment showed no additive glycemic effects. In agreement, PYY_3–36_ lacks insulinotropic properties and does not enhance insulin responses to GLP-1_7–36_ administration^64^. Therefore, Y2R agonists are thought to improve glucose tolerance by increasing peripheral insulin sensitivity as result of weight loss^65^.

## Conclusion

We report that linagliptin does not enhance anorectic and weight loss responses to Y2R agonist treatment in DIO mice. DPP-IV inhibitors may reduce circulating levels of PYY_3–36_ and NPY_3–36_, however, any negative modulatory effect on Y2R activity may likely not contribute to shape the therapeutic profile of DPP-IV inhibitors. Our study lends support to the notion that weight neutrality of DPP-IV inhibitors is determined by active GLP-1 levels being insufficiently increased to elicit satiety responses.

## Supplementary Information

Supplementary Figure 1.
