# Supplementary material for: Characterization of combined linagliptin and Y2R agonist treatment in diet-induced obese mice
Source: Sci Rep. 2021 Apr 13;11:8060. doi: 10.1038/s41598-021-87539-7 (PMC8044192; doi:10.1038/s41598-021-87539-7)
Supplement: Supplementary file 1 — Supplementary Figure 1. [file 41598_2021_87539_MOESM1_ESM.pdf]

Characterization of combined linagliptin and Y2R agonist treatment in diet-induced obese mice

Henrik H Hansen[1], Rikke V Grønlund[1], Tamara Baader-Pagler[2], Peter Haebel[2], Harald Tammen[3], Leif Kongskov Larsen[1], Jacob Jelsing[1], Niels Vrang[1], Thomas Klein[2]

[1]Gubra, Hørsholm, Denmark; [2]Department of Cardiometabolic Diseases Research, Boehringer Ingelheim Pharma GmbH & Co., Biberach, Germany; [3]PXBioVisioN, Hannover, Germany.

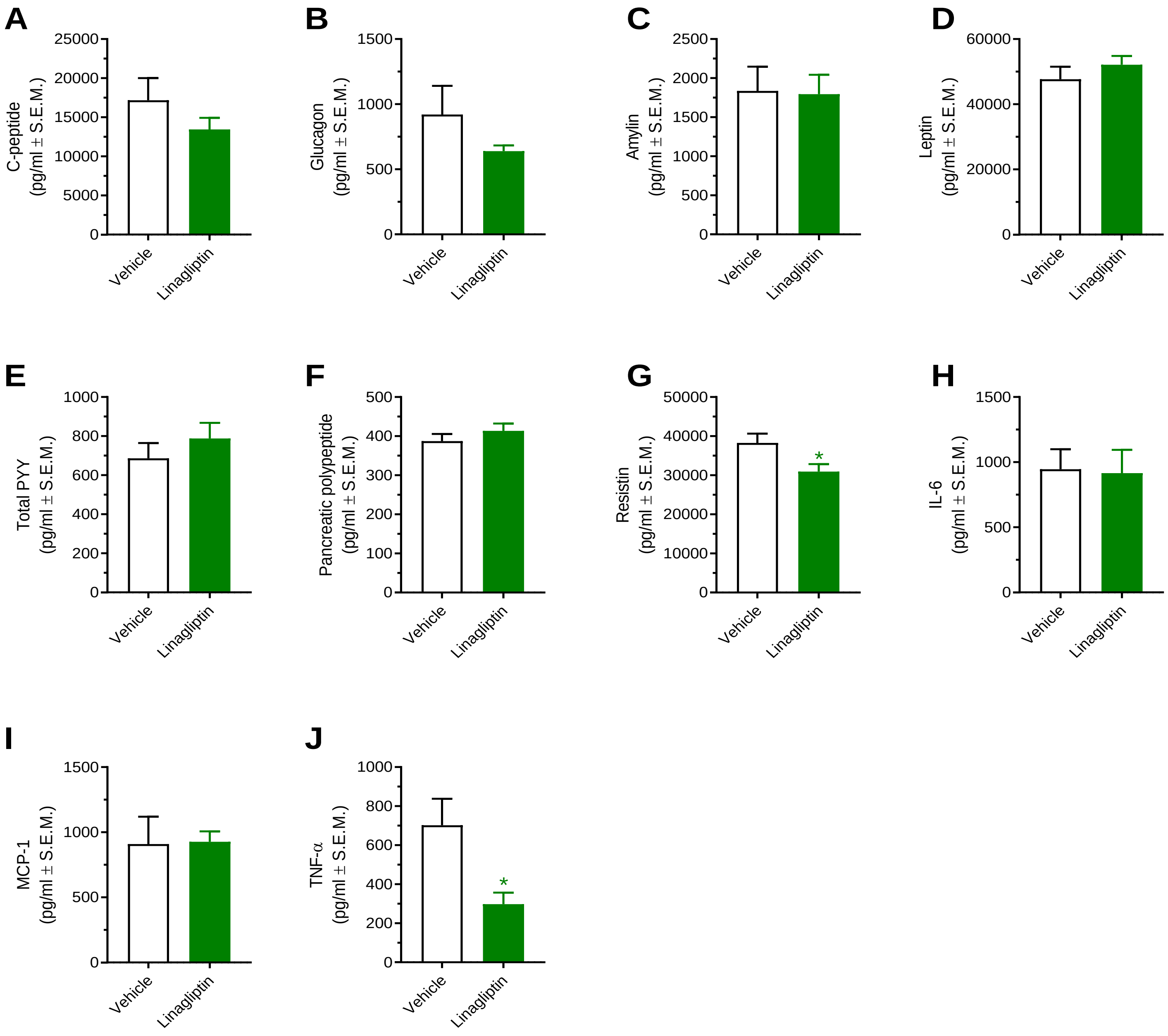

Supplementary Figure 1. Terminal (4hrs post-dosing) plasma concentrations of various metabolic markers in DIO mice following 14 days of treatment with vehicle (SC) or linagliptin (3 mg/kg/day, SC). **(A)** C-peptide, **(B)** Glucagon, **(C)** Amylin, **(D)** Leptin, **(E)** Total PYY, **(F)** Pancreatic polypeptide, **(G)** Resistin, **(H)** IL-6, **(I)** MCP-1, **(J)** TNF-α. *p<0.05 vs. vehicle control (unpaired t-test).
